# Design and synthesis of a cyclitol-derived scaffold with axial pyridyl appendages and its encapsulation of the silver(I) cation

**DOI:** 10.3762/bjoc.6.115

**Published:** 2010-10-29

**Authors:** Pierre-Marc Léo, Christophe Morin, Christian Philouze

**Affiliations:** 1 Département de Chimie Moléculaire (CNRS, UMR 5250, ICMG FR-2607) Université Joseph Fourier, 301 Rue de la Chimie, 38402 Grenoble Cedex, France

**Keywords:** cyclitol, glyco-scaffolds, hexadentate, *scyllo*-inositol, silver(I) complexation

## Abstract

Conversion of a *myo*-inositol derivative into a *scyllo*-inositol-derived scaffold with *C*_3_*_v_* symmetry bearing three axial pyridyl appendages is presented. This pre-organized hexadentate ligand allows complexation of silver(I). The crystal structure of the complex was established.

## Introduction

**Figure 1:**
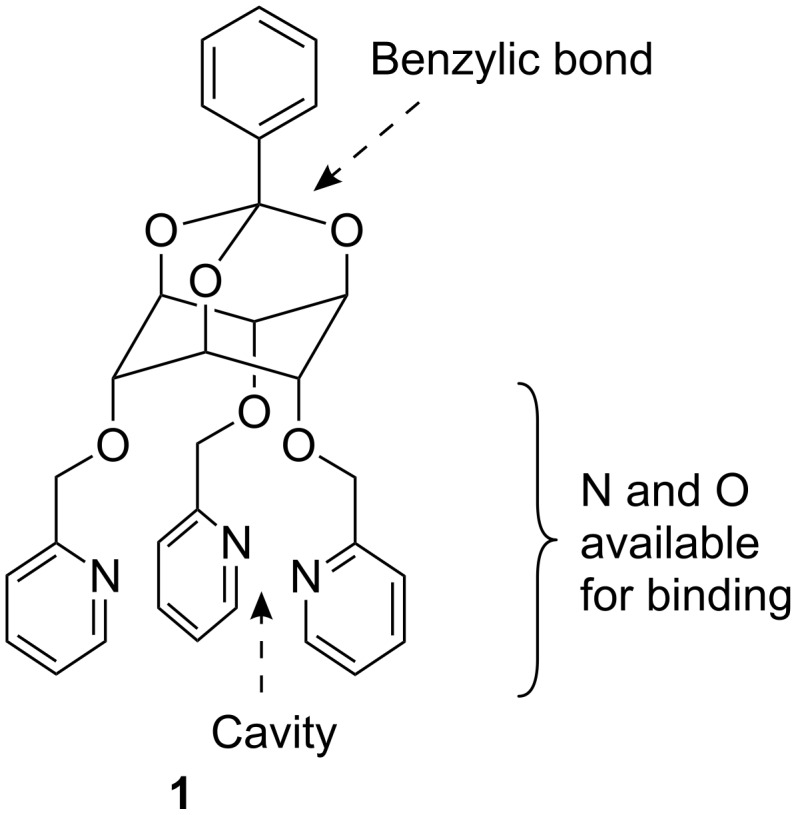
spatial representation of structure **1**.

Despite its recognized potential for nuclear medicine applications [1], the β^−^ emitter ^111^Ag (*t*_1/2_ = 7.47 d), which can be produced carrier-free and with high specific activity [2–3], has not yet found widespread uses [4–5]. This is due in part to the lack of suitable Ag complexing agents and in this work the synthesis of an cyclitol-derived scaffold designed to sequester Ag is presented. Interaction of *myo*-inositol-derived podands [6] and crown ethers [7] with Ag salts has been shown to depend on the relative spatial orientation of the binding sites moreover, in mono-orthoesters of *scyllo*-inositol the three axial hydroxy groups can be used to link substituents in a pre-organized manner [8–10]. Thus the introduction of pyridine groups (known to bind Ag(I) efficiently [11–14]) on a *scyllo*-inositol orthoester was considered, which led to the design of scaffold **1** (Figure 1). Indeed, upon complexation with participation of three oxygen atoms in the binding, no substantial rearrangement of this semi-rigid structure is to be expected. In addition, reductive cleavage of one of the ortho-ester C–H benzylic bonds, which is known to yield a free hydroxy group [15–16], would allow ligation of this scaffold.

## Results and Discussion

### Chemistry

**Figure 2:**
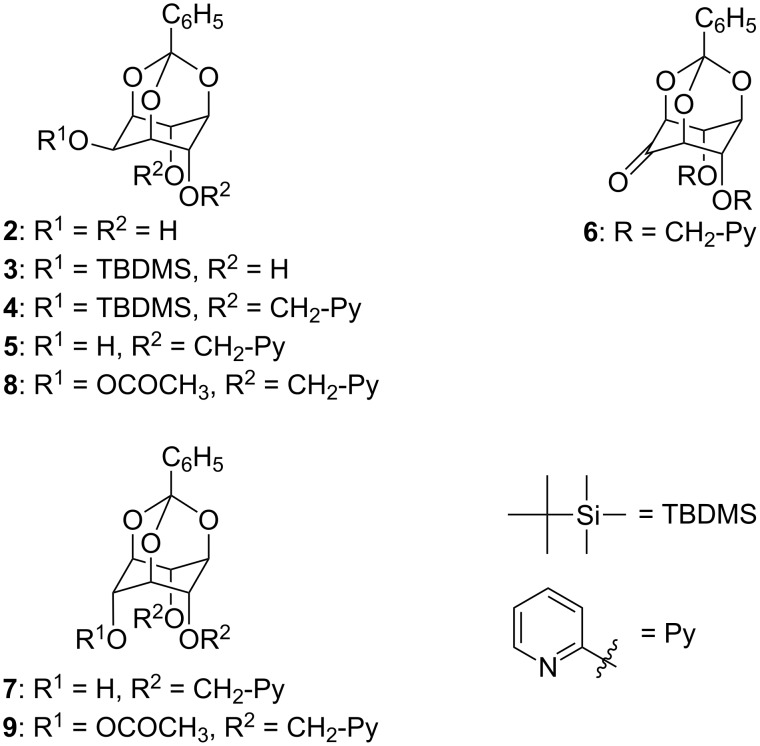
structures of compounds **2**–**9**.

The synthesis of **1** was accomplished in 6 steps from the readily available *myo*-inositol orthobenzoate (**2**) [17] (Figure 2). As *scyllo-*inositol derivatives are derived from *myo*-inositols by an oxidation/reduction sequence [8,18–19], selective protection (*tert*-butyldimethylsilyl chloride, 2,6-lutidine, DMF, 80 °C, 2 days) of the equatorial hydroxy group of **2** was first performed, to give the silyl ether **3** (70%) and subsequent picolylation (NaH, 2-picolyl chloride, DMF) of the remaining free hydroxy groups gave **4** (65%). The equatorial hydroxy group then was deblocked (tetrabutylammonium fluoride in THF) to afford **5** (86%). Inversion of configuration was accomplished via Swern oxidation [20] to furnish ketone **6** (95%), which was then reduced (NaBH_4_, CH_3_OH) to **7** (99%). Although a configurational assignment did not prove possible from ^1^H NMR data (in epimers **5** and **7** the dihedral angles of the proton geminal to the hydroxy group with vicinal protons are similar), the ^1^H- and ^13^C NMR spectra of **7** clearly differ from those of its epimer. Compounds **5** and **7** were also acetylated (to **8** and **9**, respectively) and the chemical shift of the acetyl methyl group of **9** (δ = 1.7 ppm) displayed an upfield shift compared to that of **8** (δ = 2.1 ppm) due to ring-current effects from through-space interactions with the pyridyl groups. That a *scyllo*-inositol derivative had been obtained was confirmed after the introduction of the third pyridyl group (NaH, 2-picolyl chloride). In the resulting tri-picolyl derivative **1** (71%) a *C*_3_*_v_* axis of symmetry is now present, which leads to simplification of NMR spectra. The structure of **1** was subsequently confirmed (see below) by X-ray diffraction of a single crystal of its Ag-complex.

### Complexation

Incremental addition of silver(I) trifluoromethanesulfonate to a solution of **1** in 1:1 CD_3_OD/CDCl_3_ was monitored by NMR. A single set of resonances was observed at all concentrations. That the pyridine rings and the "oxygenated cavity" were involved in silver binding was shown by the relevant shifts in both the ^1^H- and ^13^C-NMR spectra (see Supporting Information File 1). However, in the absence of characteristic signals for Ag(I)-complex (even at low temperature: −50 °C) NMR methods cannot be used for the determination of the complex stability constant. The electrospray mass spectrum (from the very solution contained in the NMR tube) revealed a 1:1 complex, with the expected ^107^Ag/^109^Ag isotopic ratio. The stability constant of the complex could be determined by spectrophotometric titration, which was carried out in 1:1 aqueous methanol (the complex is not water soluble) and its value (log *K***_1_**_·Ag_ = 3.8 ± 0.2) is in agreement with that of a previously described Ag(I) complex in a N_3_O_3_ environment [13].

**Figure 3:**
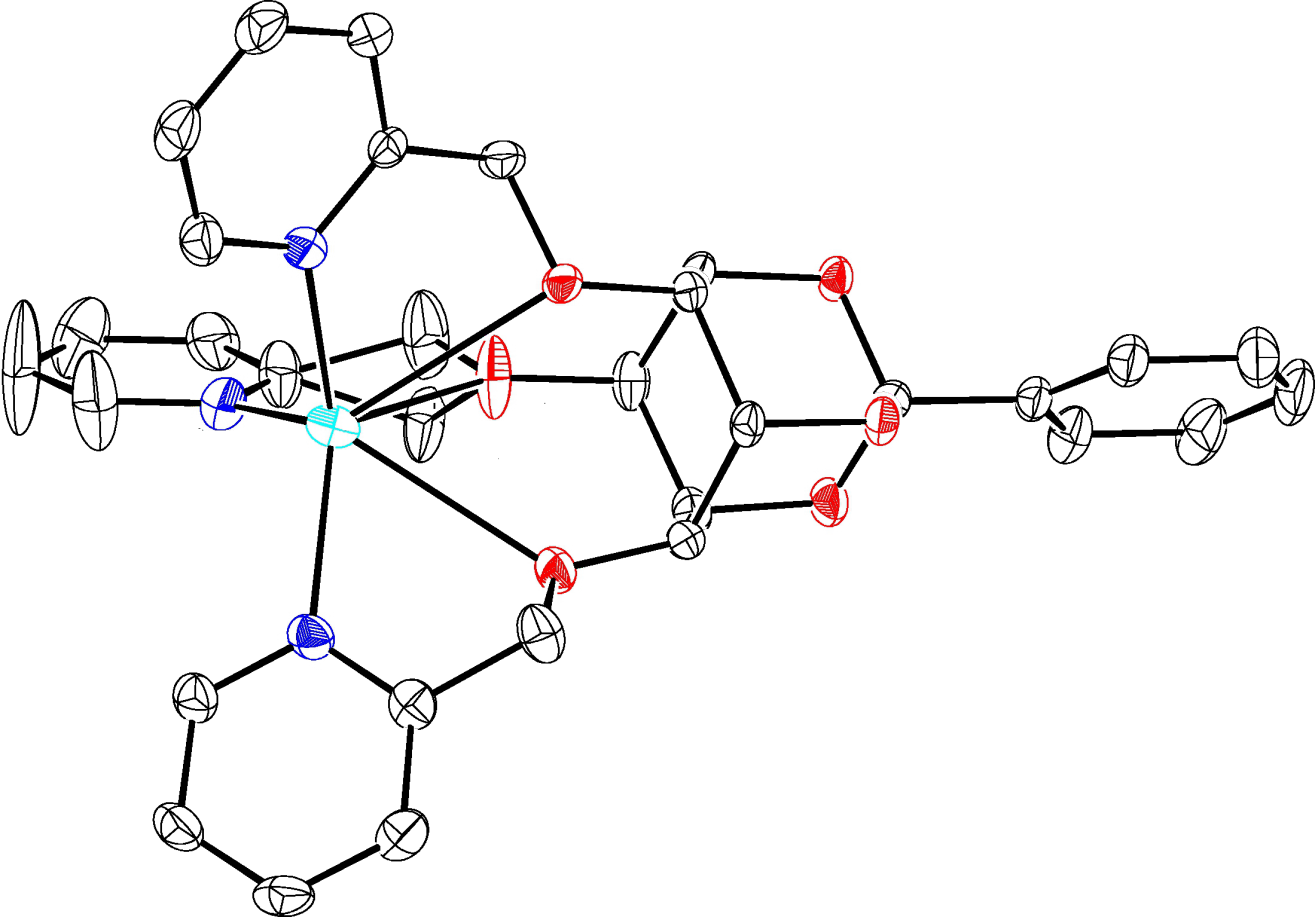
ORTEP drawing for the **1**·Ag(I) cation complex (ellipsoids are drawn at the 50% probability level and H atoms are omitted for clarity).

Study of the molecular structure of the complex was made possible as a single crystal could be grown by slow evaporation of a methanol/ethanol solution of stoichiometric amounts of **1** and silver trifluoromethanesulfonate. The complex crystallises in the triclinic system, with a pseudo-ternary axis of symmetry. Its coordination sphere involves all 3 nitrogen atoms as well as the 3 oxygen atoms which are implied in the linkage of the pyridines (Figure 3). Bond distances and angles (see Supporting Information File 1) are in accord with those of known silver complexes which are hexa-coordinated with pyridyl groups and oxygen atoms [13–14].

## Conclusion

A rigid scaffold with *C*_3_*_v_* symmetry bearing three appendages in a pre-organized manner, **1**, could be synthesized in 6 steps from *myo*-inositol orthobenzoate and in 25% overall yield. Noteworthy is that the synthetic scheme may allow variation of heterocyclic substituents, which need not be the same since they are introduced at different stages of the synthesis. The complex formed with silver cation (log *K*_1·Ag_ = 3.8 ± 0.2) was shown to involve N_3_O_3_ coordination with the Ag(I) ion lying within the cage. However, the complex is not water soluble (which precludes its use for biomedical applications) although the grafting of hydrophilic appendages to aromatic residues could increase its hydrophilicity.

## Supporting Information

Supporting Information features experimental procedures, ^1^H NMR and ^13^C NMR spectra; electrospray mass spectrum and the crystal structure of the Ag complex.

File 1 Synthesis and characterisation data for compounds **1**–**9** and **1**·Ag(I).
