# Supplementary material for: Design and synthesis of a cyclitol-derived scaffold with axial pyridyl appendages and its encapsulation of the silver(I) cation
Source: Beilstein J Org Chem. 2010 Oct 29;6:1022–4. doi: 10.3762/bjoc.6.115 (PMC2982085; doi:10.3762/bjoc.6.115)
Supplement: File 1 — Synthesis and characterisation data for compounds 1–9 and 1·Ag(I). [file Beilstein_J_Org_Chem-06-1022-s001.pdf]

Supporting Information

for

Design and synthesis of a cyclitol-derived scaffold with axial pyridyl appendages and its encapsulation of the silver(I) cation

Pierre-Marc Léo, Christophe Morin* and Christian Philouze

Address: Département de Chimie Moléculaire (CNRS, UMR 5250, ICMG FR-2607)

Université Joseph Fourier, 301 Rue de la Chimie, 38402 Grenoble Cedex, France.

Email: Christophe Morin* - Christophe.Morin@ujf-grenoble.fr

*Corresponding author

Synthesis and characterisation data for compounds 1–9 and 1·Ag(I).

Pages S1–S3: Synthetic procedures.

Page S3: Determination of stability constant

Pages S3–S5: Radiocrystallographic analysis of Ag complex.

Pages S6–S7: Electrospray mass spectrum of Ag complex

Pages S8–S21: 300 MHz ^{1}H NMR and 75 MHz ^{13}C NMR spectra.

Synthetic procedures

2-Picolyl chloride. A solution of sodium bicarbonate (3 g) in water (50 mL) was added with stirring to 2-picolyl chloride hydrochloride (3 g, 18.3 mmol) (caution: foaming). When the foaming had subsided, the mixture was extracted with diethyl ether and the organic layer washed with water and dried (sodium sulfate). After evaporation of the ether a red residue (which can be stored under argon in the cold) was obtained. Purification was carried out immediately before use by column chromatography on basic alumina (activity I) eluting with diethyl ether.

2-*O*-*tert*-Butyldimethylsilyl-*myo*-inositol-1,3,5-orthobenzoate (**3**) [procedure adapted from S. Angyal, *Carbohydr. Res*. **2000**, *325,* 313-320]. Under an argon atmosphere, 2,6-lutidine (1.8 g, 42 mmol) followed by *tert*-butyldimethylsilyl chloride (1.0 g, 29.75 mmol) was added to a solution of *myo*-inositol orthophenylacetate (**2**) [Bhosekar, G., Murali, C, Gonnade, R.G., Shashidar, M.S., Bhadbhade, M.M. *Cryst. Growth Des.* **2005**, *5,* 1977-1982] (1.75 g, 17.5 mmol) in dry DMF (14 mL). After 2 day, the mixture was concentrated to half-volume (bath temperature 80 °C) and water (10 mL) added. On cooling and filtration **3** was obtained, (1.75 g, 70%).

2-*O*-*tert*-Butyldimethylsilyl-4,6-di-*O*-(2-picolyl)-*myo*-inositol-1,3,5-orthobenzoate (**4**). At 0 °C, to a stirred solution of **3** (1.52 g, 4 mmol) in dry DMF (10 mL) was added sodium hydride (0.35 g of a 60% dispersion in mineral oil, 8.8 mmol). When evolution of gas had ceased, freshly prepared 2-picolyl chloride (2.55 g, 20 mmol) and a catalytic quantity of sodium iodide were added. After overnight stirring (with protection from light), water was added and the reaction mixture extracted with 1:1 diethyl ether/pentane. The organic layer was dried, concentrated and the residue purified by column chromatography on silica gel (elution with 97:3 dichloromethane/methanol) to give **4** (1.63 g, 65%).

2,4-Di-*O*-(2-picolyl)-*myo*-inositol-1,3,5-orthobenzoate (**5**). Under an argon atmosphere, a 1 *M* solution of tetrabutylammonium fluoride in THF (4.5 mL, 4.5 mmol) was added *via* syringe to a solution of **4** (1.58 g, 2.81 mmol) in dry THF (7 mL). After 3 h stirring, water was added and the reaction mixture extracted with dichloromethane. After drying and evaporation of the volatiles, column chromatography on silica gel (elution with 95:5 dichloromethane/methanol) gave **5** (1.03 g, 86%).

4,6-Di-*O*-(2-picolyl)-*myo/scyllo*-inos-2-ose-1,3,5-orthobenzoate (**6**). Freshly distilled oxalyl chloride (0.76 g, 5.98 mmol) was added to dry dichloromethane (2.5 mL) and the solution stirred under an argon atmosphere at -70 °C. Dry DMSO (0.75 mL) was cautiously added dropwise, followed after 5 min by the dropwise addition of a solution of **5** (0.8 g, 1.79 mmol) in dichloromethane (2.5 mL). The solution was stirred at -30 °C for 1 h and then cooled to -70 °C before the addition of dry diethylisopropylamine (4 mL) . The cooling bath was removed and after 3 h water was added. The mixture was extracted with dichloromethane, dried and the concentrated. The residue was purified by column chromatography on silica gel (elution with 95:5 dichloromethane/methanol) to afford **6** (0.75 g, 95%).

2,4-Di-*O*-(2-picolyl)-*scyllo*-inositol-1,3,5-orthobenzoate (**7**). Sodium borohydride (0.75 g) was added portionwise to a stirred solution of **6** (0.75 g, 1.68 mmol) in methanol (60 mL) at 0 °C (caution: foaming). After 30 min, acetone (1 mL) was added and the volatiles were removed under reduced pressure. Co-evaporation of the residue with methanol was carried out twice before the addition of water. The residue was extracted with dichloromethane and dried. Removal of the solvent afforded **7** (0.76 g, 99%).

2-*O*-Acetyl-4,6-di-*O*-(2-picolyl)-*myo*-inositol-1,3,5-orthobenzoate (**8**) and **2-*O*-acetyl-4,6-di-*O*-(2-picolyl)-*scyllo*-inositol-1,3,5-orthobenzoate** (**9**) were prepared from **5** and **7** respectively by standard methods (Ac_2O , pyridine, RT, overnight).

2,4,6-Tri-*O*-(2-picolyl)-*scyllo*-inositol-1,3,5-orthobenzoate (**1**). A solution of **7** (0.63 g, 1.40 mmol) in dry DMF (6 mL) was stirred at 0 °C and sodium hydride (0.15 g of a 60% dispersion in mineral oil, 3.9 mmol) added. When evolution of gas had ceased, freshly prepared 2-picolyl chloride (0.54 g, 4.24 mmol) was added and stirring continued for 3 h.

After hydrolysis, the mixture was extracted with 1:1 diethyl ether/pentane. The organic layer was dried, concentrated and purified by column chromatography on silica gel (elution with 95:5 dichloromethane/methanol) to afford **1** (0.54 g, 71%).

Silver(I) 2,4,6-tri-*O*-(2-picolyl)-*scyllo*-inositol-1,3,5-orthobenzoato trifluoro-methanesulfonate (**1**·$AgSO_3CF_3$) Silver(I) trifluoromethanesulfonate (19.020 mg, 35.6 μmol) and **1** (9.151 mg, 35.6 μmol) were dissolved in hot ethanol (1 mL) and after some cooling, methanol (1 mL) was added. A cotton plug was placed on top of vial to allow slow evaporation of the solvents, which led to crystals suitable for cristallographic analysis. Mp: 170 °C (softens), 190 °C (fully melts). IR (film): ν 2878 (w), 1601 (s), 1574 (w), 1371 (s), 1305 (s), 1027 (m), 761 (s) cm^{-1}.
Crystallographic data: pp: S3–S5; Electrospray MS: pp S6–S7; NMR spectra: pp S8 and S10.

Determination of the stability constant

Spectrophotometric titration of **1** (1.96 10^{-4} M) with a solution of $AgSO_3CF_3$ (2.135 10^{-3} M) was carried out at 25°C in H_2O:MeOH (1:1) on a Varian Cary 50 UV–visible spectrophotometer equipped with a Peltier thermostating accessory. The absorbance data were processed with the SPECFIT program. The best fit was obtained by considering the formation of 1:1 complex.

Radiocrystallographic analysis

Crystal mounting and data collection: a prismatic crystal (0.40 x 0.30 x 0.07 mm) was selected and mounted on a glass fiber using epoxy resin. Recording of data was performed at 200 °K using a Bruker-AXS-Enraf-Nonius kappa CCD diffractometer with monochromatic (graphite) MoKα radiation. EVAL software was used for cell-unit determination, refinement and data reduction. The data were corrected for decays and for Lorentz and polarization effects. The results are summarised in the following table.

Table: Crystal Data and Structure Refinement

Name	Silver(I) 2,4,6-tri-O-(2-picolyl)-*scyllo*-inositol-1,3,5-orthobenzoato trifluoromethanesulfonate
CCDC deposit no.	CCDC 782357
Color/shape	Colorless/prism
Chemical formula	$C_{32}H_{29}AgF_3N_3O_9S$
Formula weight	796.52
Temperature, K	200
Crystal system	triclinic
Space group	P-1
Unit-cell dimensions	a = 11.452(2) Å<br>b = 11.676(3) Å<br>c = 14.389(2)) Å<br>α = 98.00(2) °<br>β = 105.78(2) °<br>γ = 118.07(2) °
Unit-cell volume (Å^3)	1550.8(9)
Z	2
Density (calculated), g/cm^3	1.706

Absorption coefficient, mm^{-1}	0.796
Diffractometer/scans	Bruker-Enraf-Nonius kappaCCD/phi and omega
Type of radiation	Mo ($K\alpha$) :0.71073 Å
θ range for data collection, (°)	1.55-25
Reflections measured	16528
Independent/observed reflections	5495/4741 [$I>2\sigma(I)$]
Data/restraints/parameters	4741/0/451
Goodness of fit on F	1.734
Final R indices [$I>2\sigma(I)$]	R1 = 0.0349, wR2 = 0.0470

Crystal structure determination, refinements and discussion. The structure was solved using direct methods from SIR-92 [Altomare A, Cascarano G, Giacovazzo C, Guagliardi A *J Appl Cryst* **1993**, *26*, 343]; C, Ag ,F, N, O, S, atoms were refined anisotropically by the full matrix least-squares method and H atoms were calculated from idealised positions. Drawing of the molecular structure was realised with the use of ORTEP [Johnson CK (**1976**) ORTEPII. Report ORNL-5138. Oak Ridge National Laboratotry, Oak Ridge, Tennessee, USA].

The silver complex (**1**·$AgSO_2CF_3$) crystallises in the triclinic system which contains few symmetry operations. The complex displays a pseudo ternary axis passing through the silver cation and the C6 C7 bond. This is a pseudo ternary axis however, as one of the pyridyl groups (bearing N3) does not obey symmetry due to slight statistical disorder as evidenced by the two possible positions of C26 atom which is spread over two different positions (C26a and C26b). This disorder may cause two slightly different orientations of the pyridine group, which are too close however to be refined separately (hence the unusual ellipsoid at C30). As a consequence a pseudo mirror plane passing through the pseudo ternary axis and containing the slightly disordered pyridyl group can be observed .

Table 2. Selected bond distances and angles

Bond (Å)	
Ag1 O4	2.812(2)
Ag1 O5	2.716(1)
Ag1 O6	2.778(2)
Ag1 N1	2.250(2)
Ag1 N2	2.268(2)
Ag1 N3	2.313(2)
Angles (°)	
O4 Ag1 O5	65.72(5)
O4 Ag1 O6	63.32(5)
O4 Ag1 N1	65.37(5)
O4 Ag1 N2	121.61(6)
O4 Ag1 N3	120.54(6)
O5 Ag1 O6	64.27(5)
O5 Ag1 N1	124.88(6)
O5 Ag1 N2	65.23(6)
O5 Ag1 N3	114.89(6)
O6 Ag1 N1	112.20(6)
O6 Ag1 N2	117.53(6)
O6 Ag1 N3	65.57(6)
N1 Ag1 N2	126.07(6)
N1 Ag1 N3	110.82(7)
N2 Ag1 N3	107.61(8)

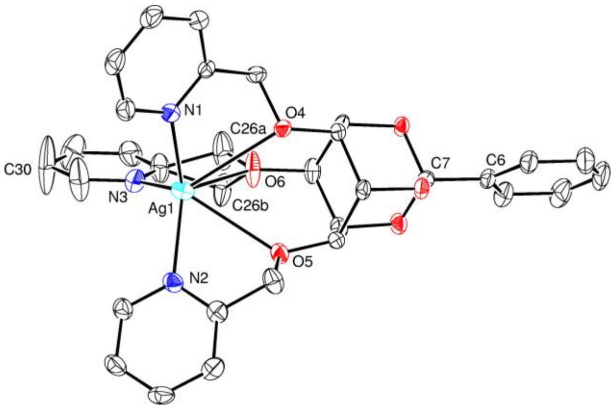

Figure 1: ORTEP drawing for the Ag(I) cation complex. H atoms were omitted for clarity. Ellipsoids were drawn at the 50% probability level.

Display Report

Analysis Info

Analysis Name	D:\Data\Serco\Christophe Morin_1_01_4828.d	Acquisition Date	3/12/2010 9:56:33 AM
Method	09_07_2008_passeur+masse target mass=600_4828.m	Operator	Laure
Sample Name	Christophe Morin	Instrument	esquire3000 plus
Comment	CM3977(Ag)<br>510-4 Mol ds CDCl3+CD3OD dilue 1/100 eau/MeOH/CH2Cl2		

Acquisition Parameter

Ion Source Type	ESI	Ion Polarity	Positive	Alternating Ion Polarity	off
Mass Range Mode	Std/Normal	Scan Begin	50 m/z	Scan End	1300 m/z
Capillary Exit	128.3 Volt	Skim 1	40.0 Volt	Trap Drive	71.5
Accumulation Time	652 µs	Averages	20 Spectra	Auto MS/MS	off

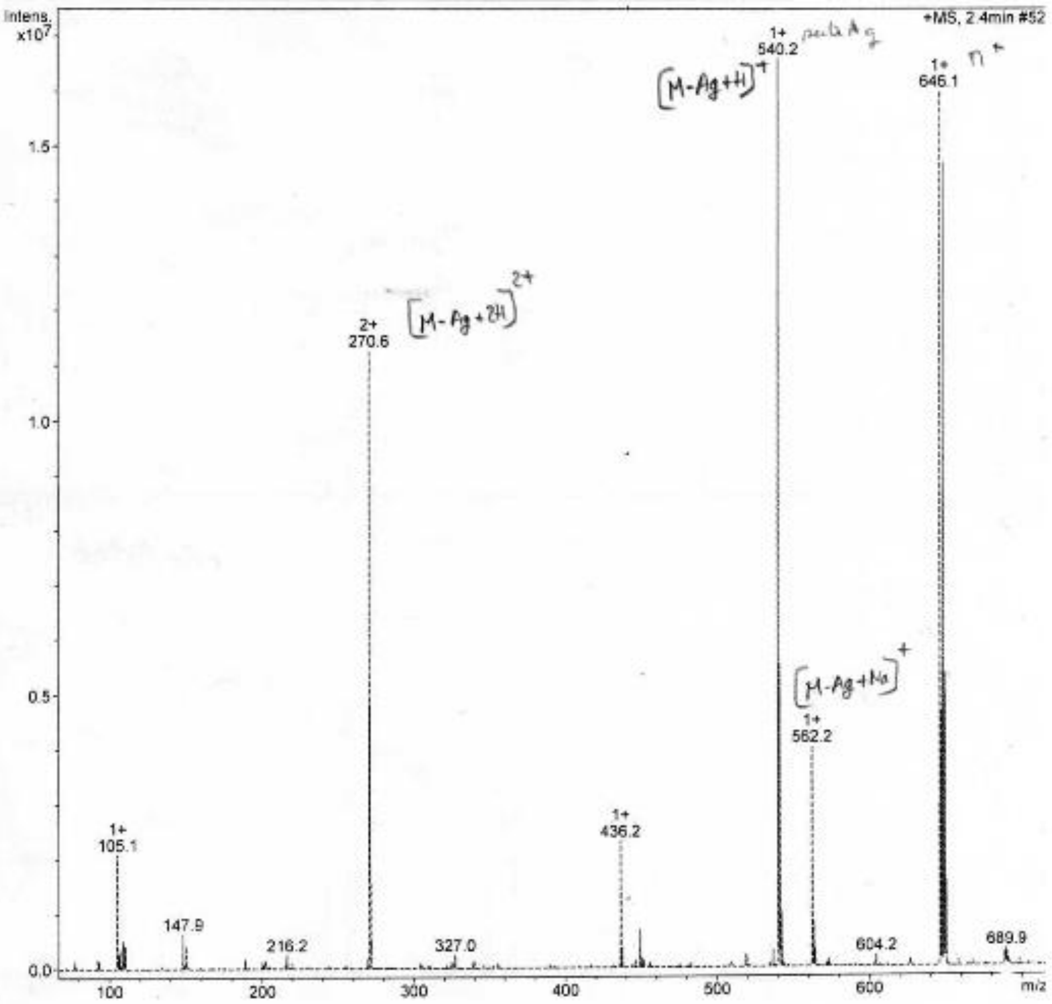

Bruker Daltonics DataAnalysis 3.3 printed: 3/12/2010 10:11:20 AM Page 1 of 1

1 with $AgOSO_2CF_3$

Display Report

Analysis Info

Analysis Name	D:\Data\Serco\Christophe Morin_1_01_4828.d
Method	09_07_2008_passeur+masse target mass=600_4828.m
Sample Name	Christophe Morin
Comment	CM3977(Ag) 510-4 Mol ds CDCl3+CD3OD dilue 1/100 eau/MeOH/CH2Cl2

Acquisition Date	3/12/2010 9:56:33 AM
Operator	Laure
Instrument	esquire3000 plus

Acquisition Parameter

Ion Source Type	ESI	Ion Polarity	Positive	Alternating Ion Polarity	off
Mass Range Mode	Std/Normal	Scan Begin	50 m/z	Scan End	1300 m/z
Capillary Exit	128.3 Volt	Skim 1	40.0 Volt	Trap Drive	71.5
Accumulation Time	652 µs	Averages	20 Spectra	Auto MS/MS	off

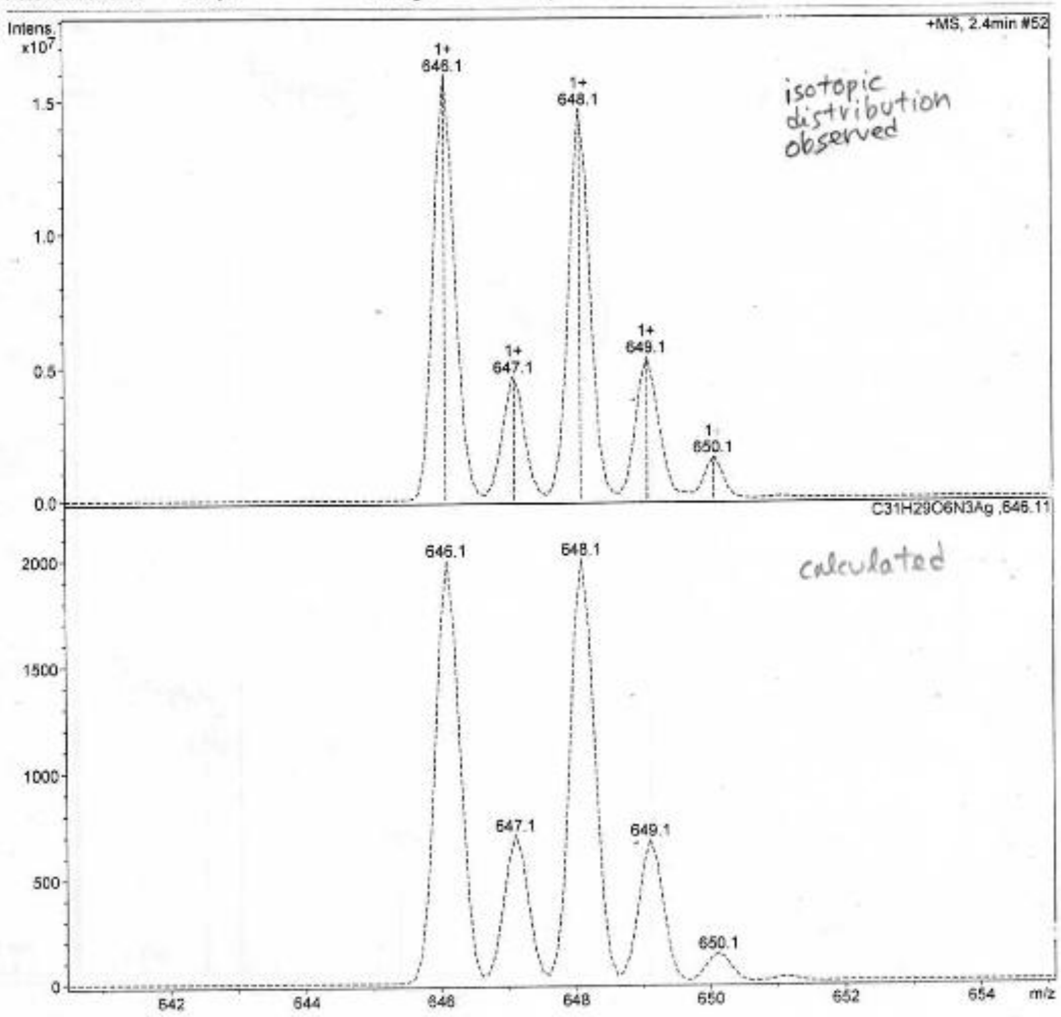

Bruker Daltonics DataAnalysis 3.3 printed: 3/12/2010 10:10:47 AM Page 1 of 1

1 with $AgOSO_2CF_3$

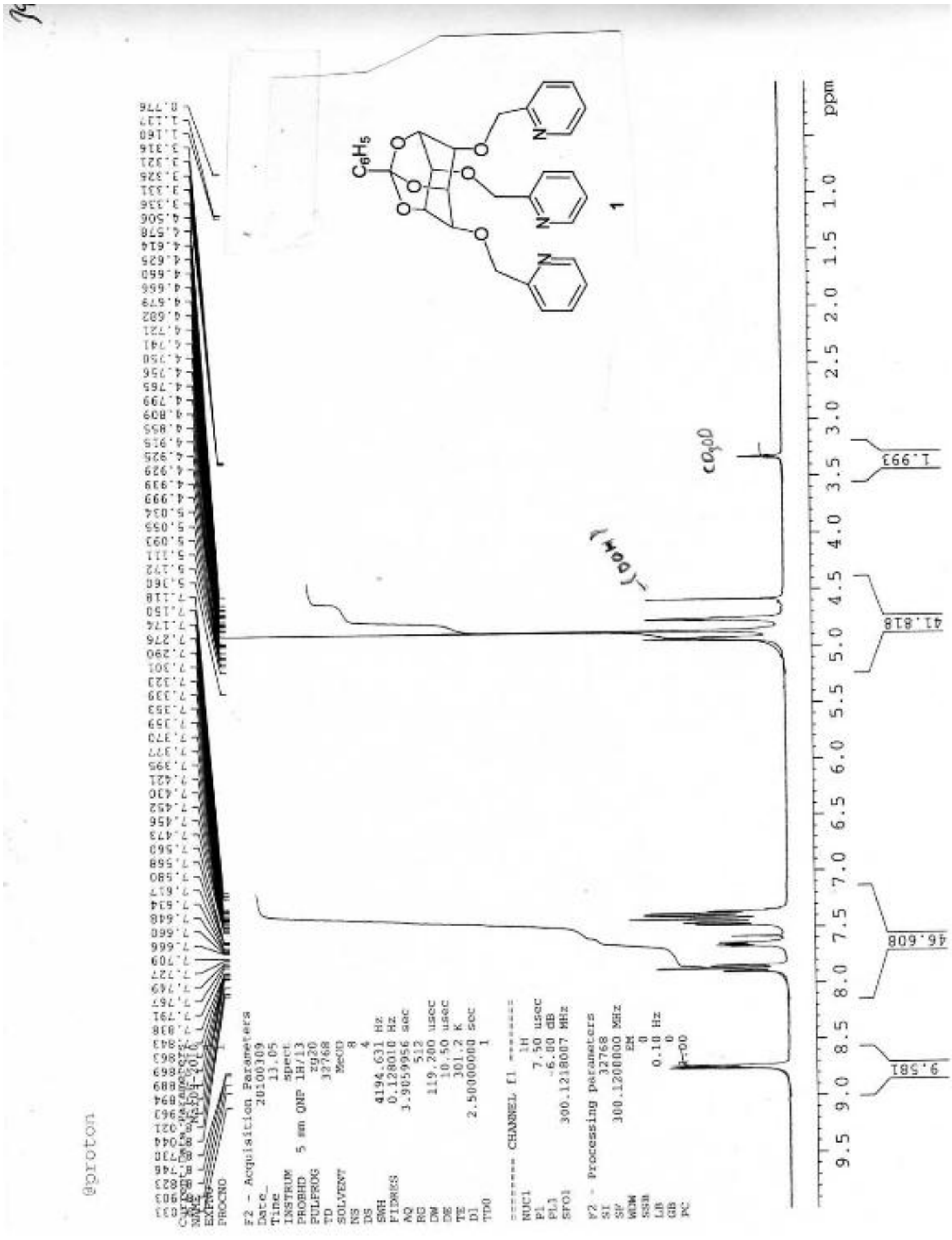

@proton
C_6H_5
1
ppm
1.993
41.818
46.608
9.581

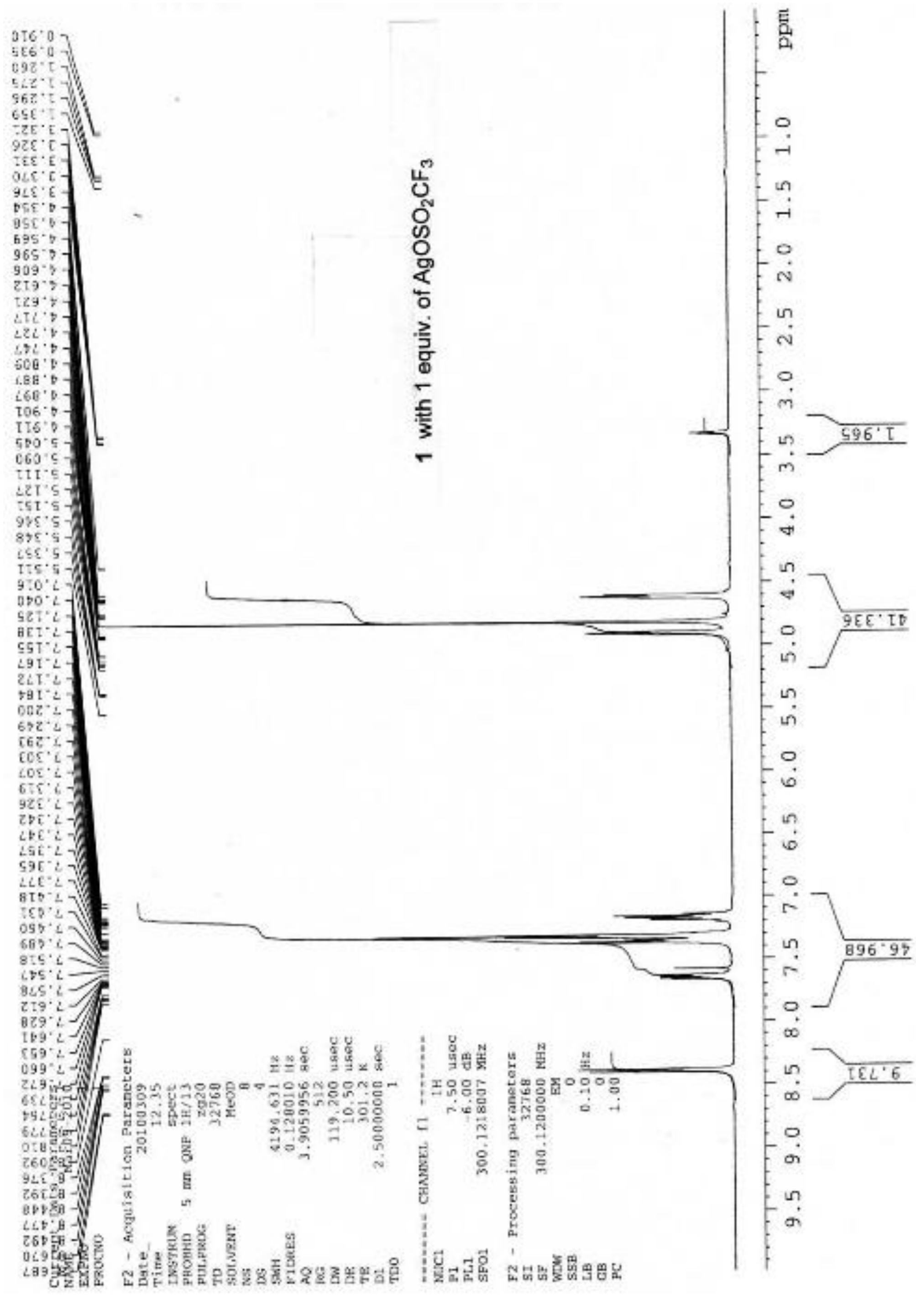
1 with 1 equiv. of $AgOSO_2CF_3$
1.965
41.336
46.968
9.731
ppm

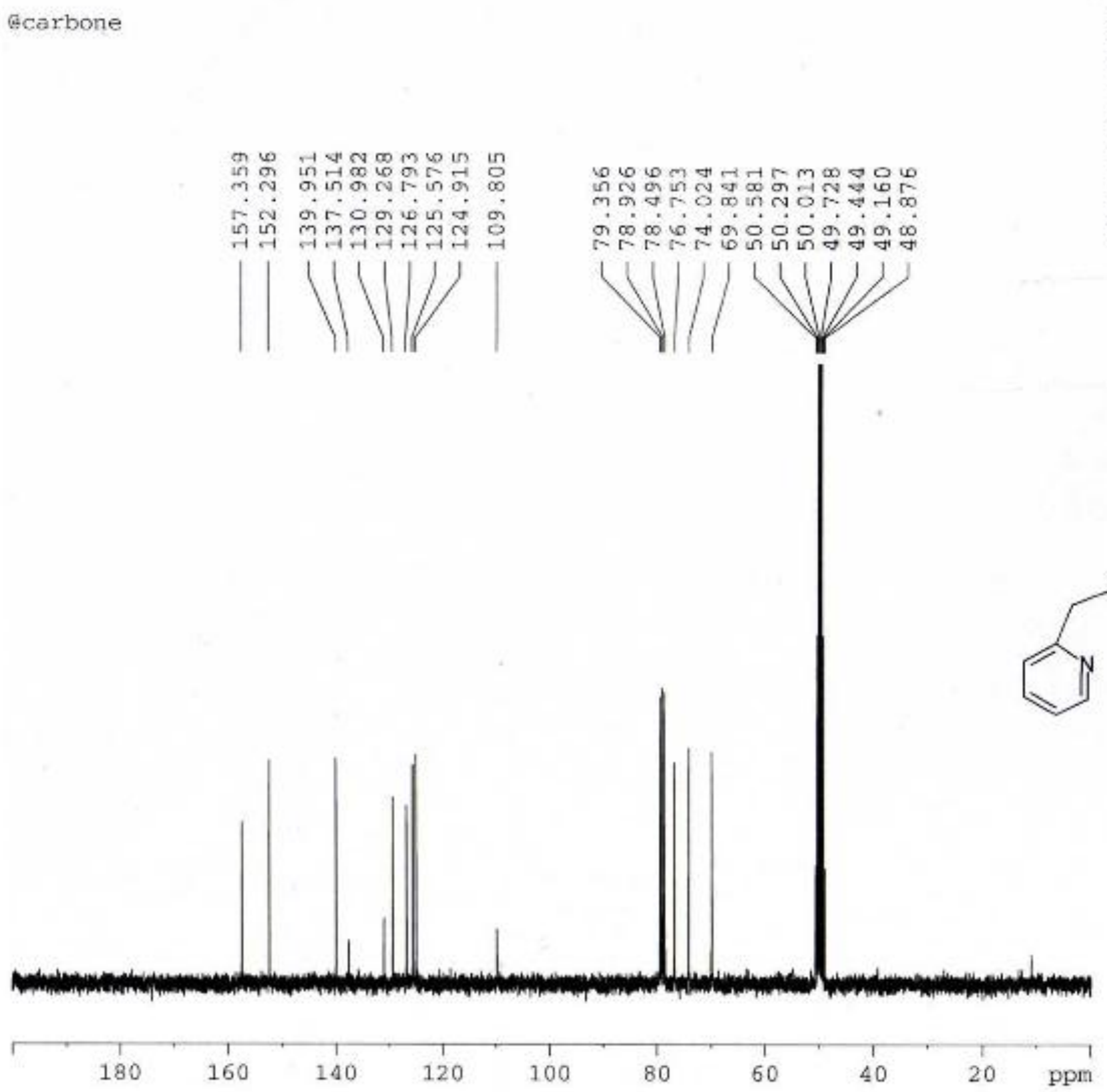

Current Data Parameters
NAME Mar09-2010
EXPNO 7
PROCNO 1

F2 - Acquisition Parameters
Date_ 20100309
Time 13.31
INSTRUM spect
PROBHD 5 mm QNP 1H/13
PULPROG zgpg30
TD 32768
SOLVENT MeOD
NS 415

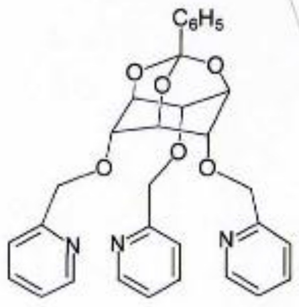

1

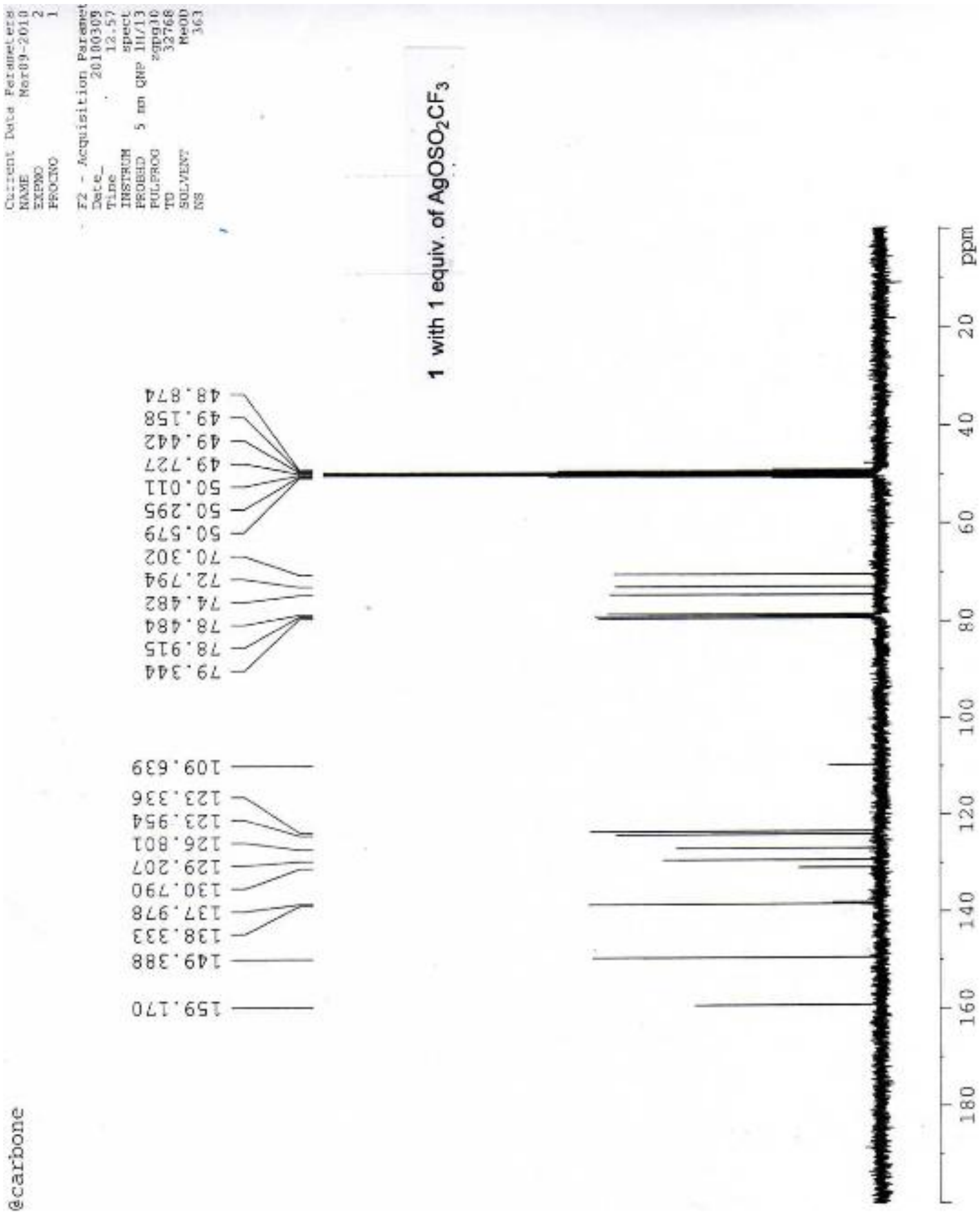
Current Data Parameters
NAME Mar09-2010
EXPNO 2
PROCNO 1
F2 - Acquisition Paramet
Date_ 20100309
Time 12.57
INSTRUM spect
PROBHD 5 mm QNP 1H/13
PULPROG zgpg30
TD 32768
SOLVENT MeOD
NS 363
1 with 1 equiv. of AgOSO2CF3
@carbone
48.874
49.158
49.442
49.727
50.011
50.295
50.579
70.302
72.794
74.482
78.484
78.915
79.344
109.639
123.336
123.954
126.801
129.207
130.790
137.978
138.333
149.388
159.170
ppm
20
40
60
80
100
120
140
160
180

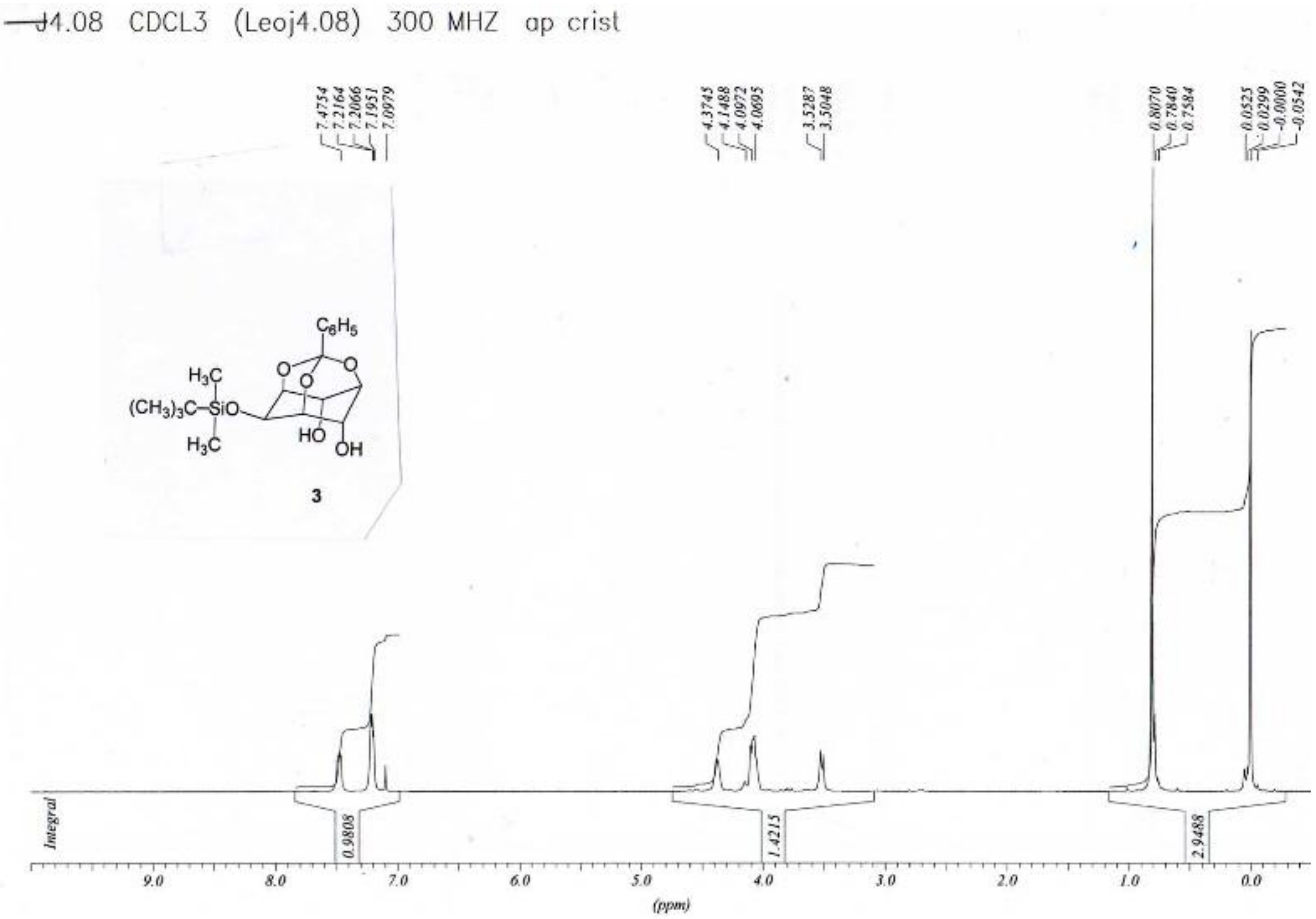
J4.08 CDCL3 (Leoj4.08) 300 MHZ ap crist
7.4754
7.2164
7.2066
7.1951
7.0979
4.3745
4.1488
4.0972
4.0695
3.5287
3.5048
0.8070
0.7840
0.7584
0.0525
0.0299
-0.0000
-0.0542
C6H5
H3C
(CH3)3C–SiO
H3C
HO
OH
3
Integral
0.9808
1.4215
2.9488
9.0
8.0
7.0
6.0
5.0
4.0
3.0
2.0
1.0
0.0
(ppm)

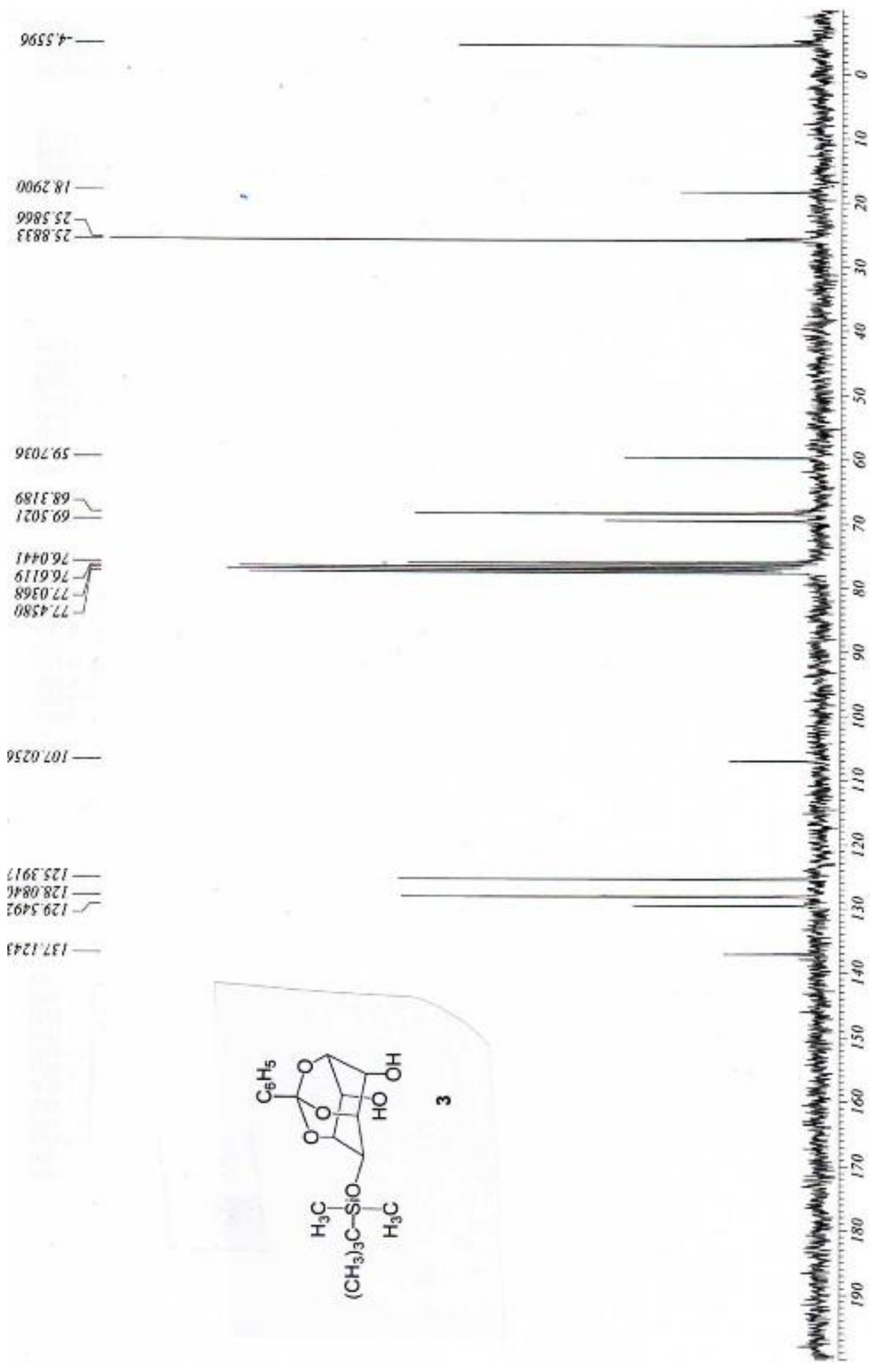
-4.5596
18.2900
25.5866
25.8833
59.7036
68.3189
69.5021
76.0441
76.6119
77.0368
77.4580
107.0256
125.3917
128.0846
129.5492
137.1243
(ppm)
3

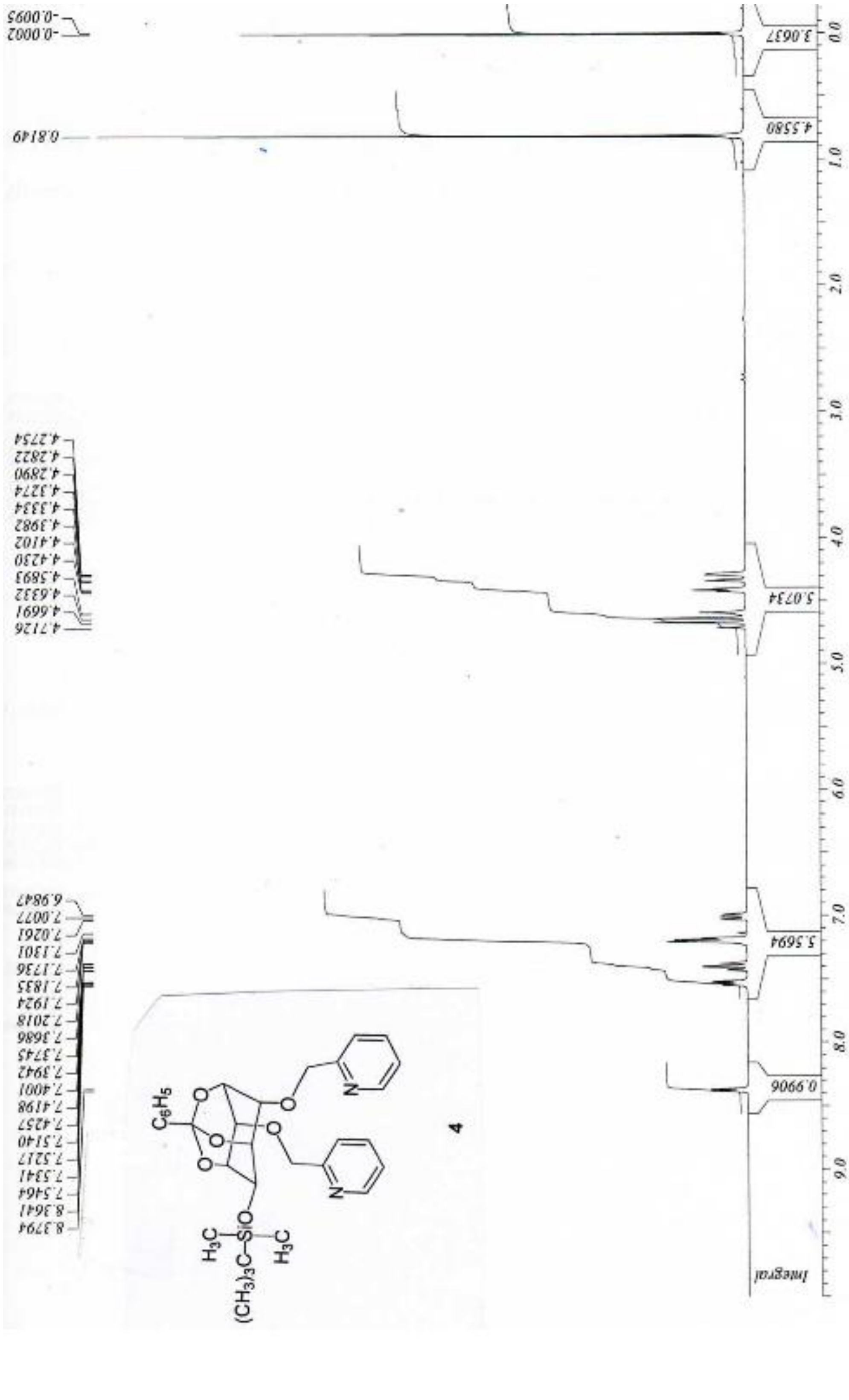
-0.0095
-0.0002
0.8149
4.2754
4.2822
4.2890
4.3274
4.3334
4.3982
4.4102
4.4230
4.5893
4.6332
4.6691
4.7126
6.9847
7.0077
7.0261
7.1301
7.1736
7.1835
7.1924
7.2018
7.3686
7.3745
7.3942
7.4001
7.4198
7.4257
7.5140
7.5217
7.5341
7.5464
8.3641
8.3794
3.0637
4.5580
5.0734
5.5694
0.9906
Integral
(ppm)
C6H5
(CH3)3C–SiO
H3C
H3C
N
N
4

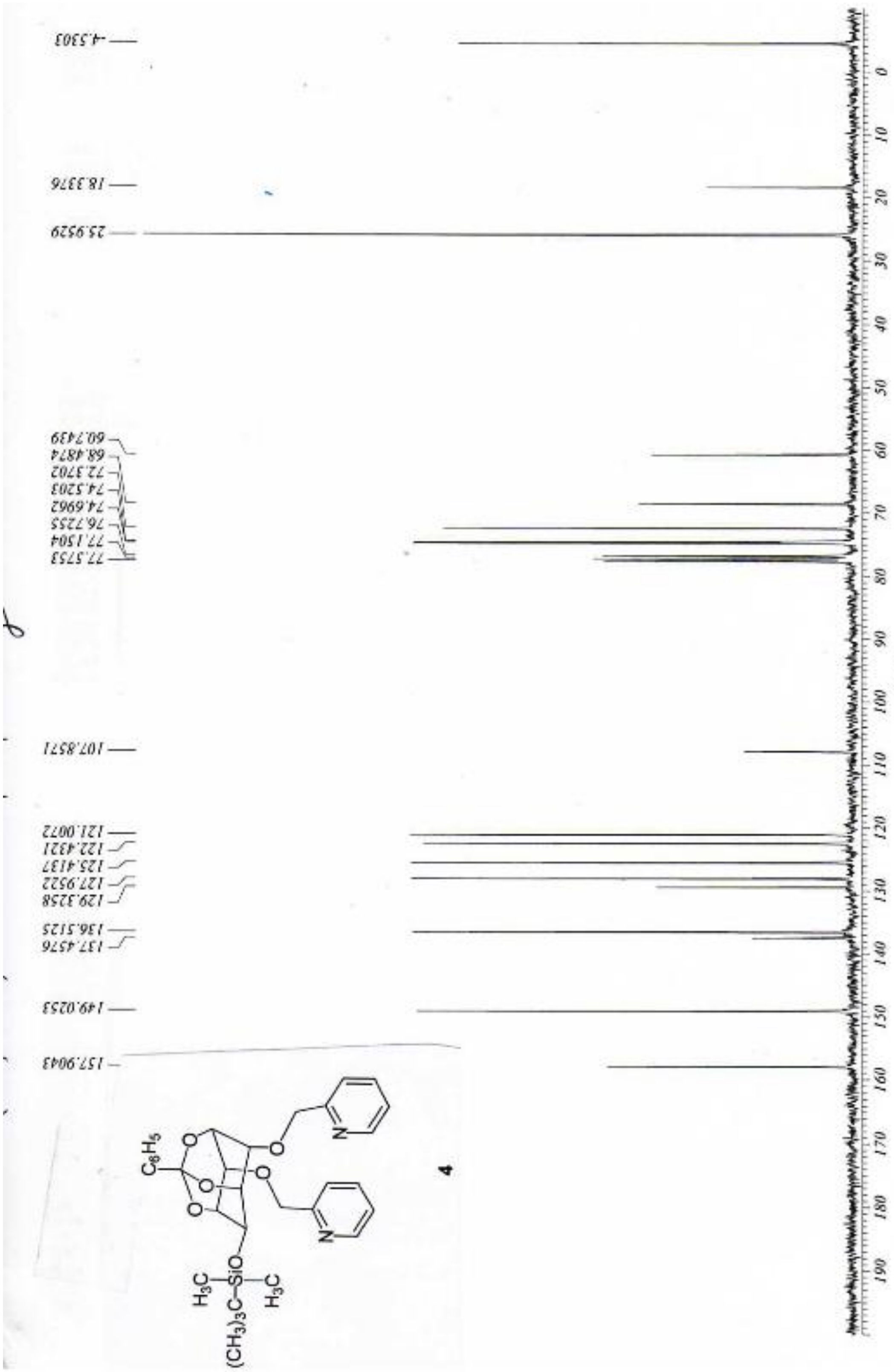
-4.5303
18.3376
25.9529
60.7439
68.4874
72.3702
74.5203
74.6962
76.7255
77.1504
77.5753
107.8571
121.0072
122.4321
125.4137
127.9522
129.3258
136.5125
137.4576
149.0253
157.9043
(ppm)
C6H5
(CH3)3C-SiO
H3C
N
4

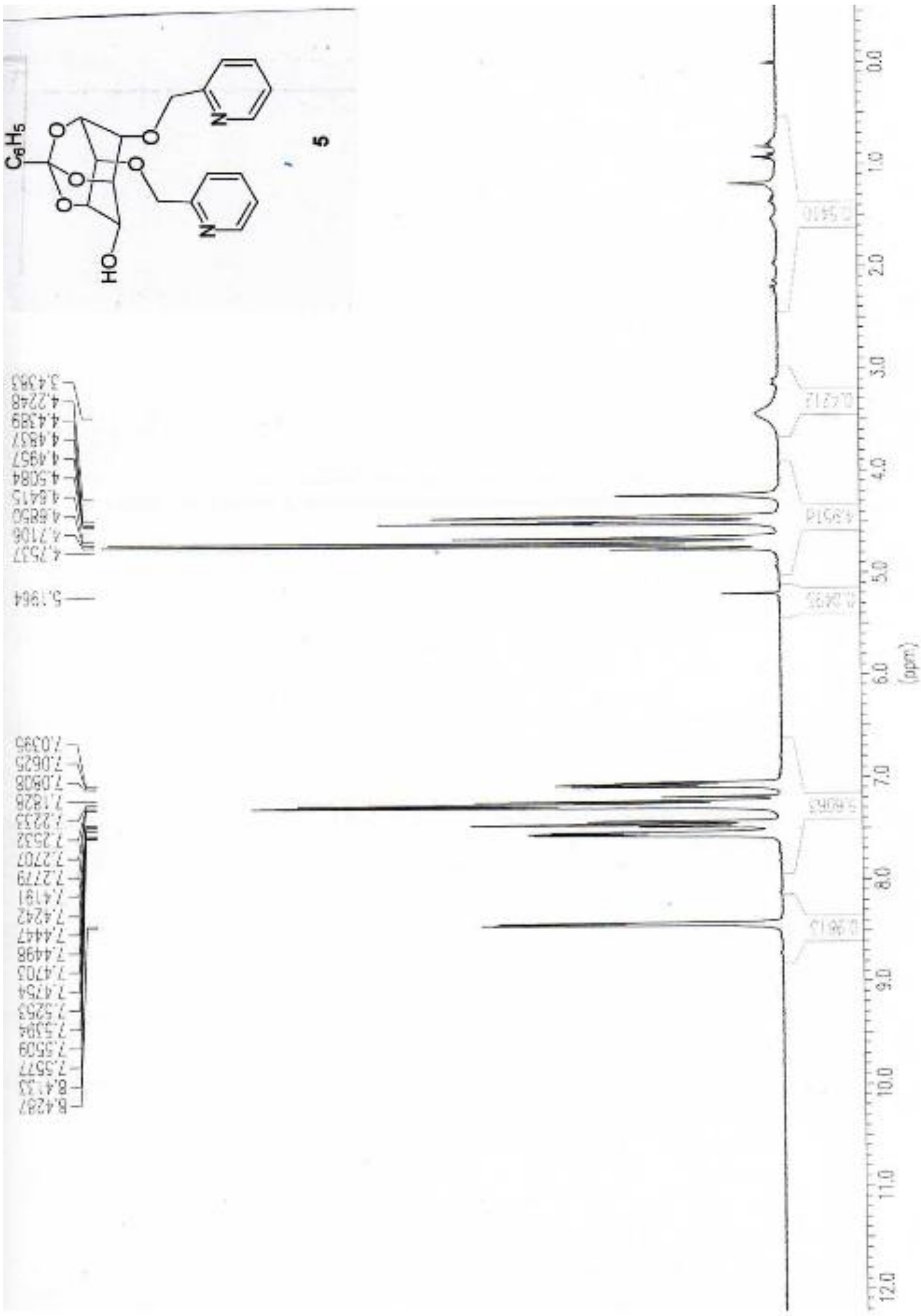
C6H5
HO
N
5
3.4383
4.2248
4.4389
4.4837
4.4957
4.5084
4.6415
4.6850
4.7106
4.7537
5.1964
7.0395
7.0625
7.0808
7.1828
7.2233
7.2532
7.2707
7.2779
7.4191
7.4242
7.4447
7.4498
7.4703
7.4754
7.5253
7.5394
7.5509
7.5577
8.4133
8.4287
0.0
1.0
2.0
3.0
4.0
5.0
6.0
7.0
8.0
9.0
10.0
11.0
12.0
(ppm)

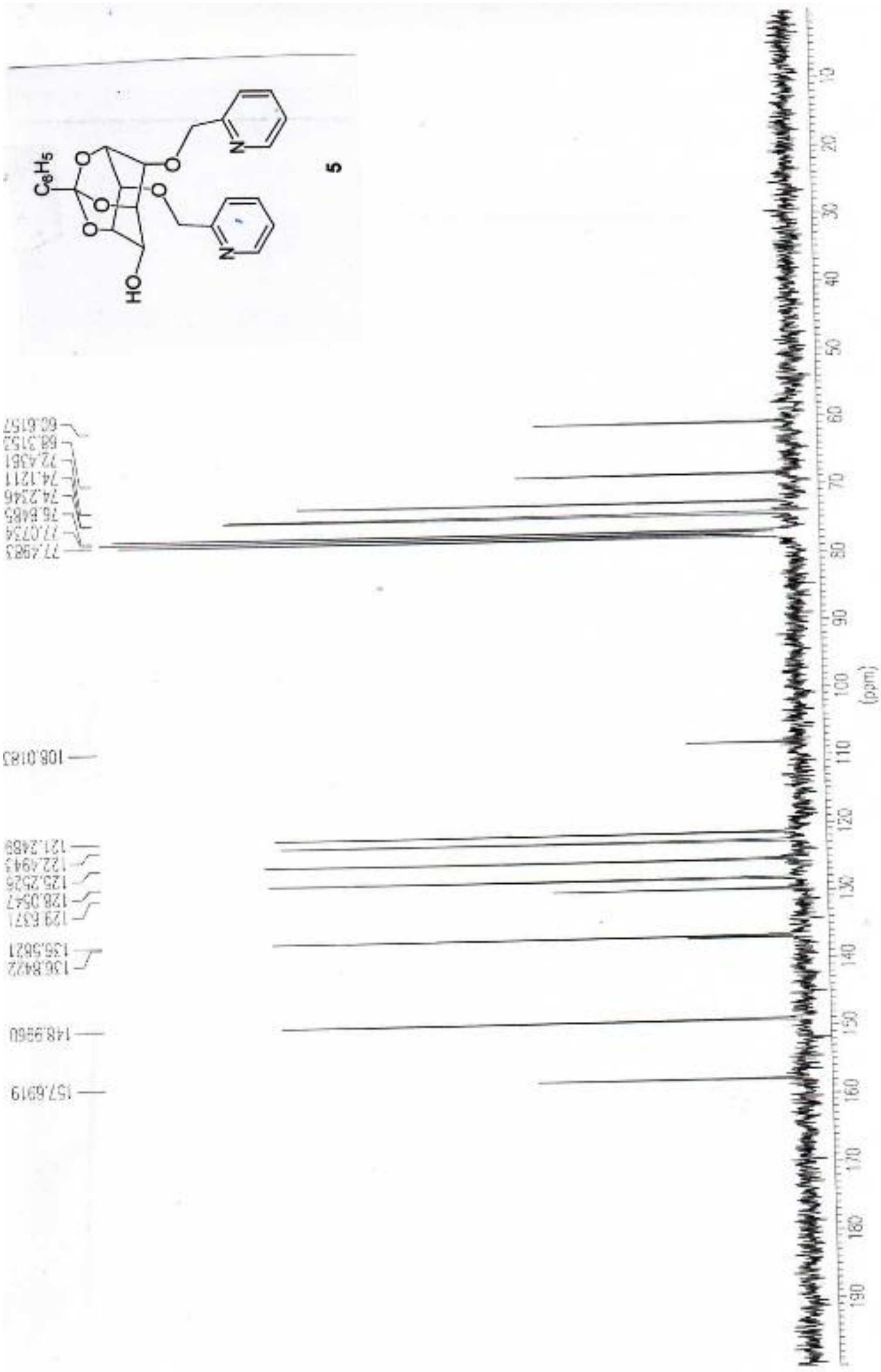
C6H5
HO
5
60.6157
68.3152
72.4361
74.1211
74.2346
76.6485
77.0734
77.4983
108.0183
121.2469
122.4943
125.2526
128.0547
129.6371
135.5621
136.8422
148.9960
157.6919
(ppm)

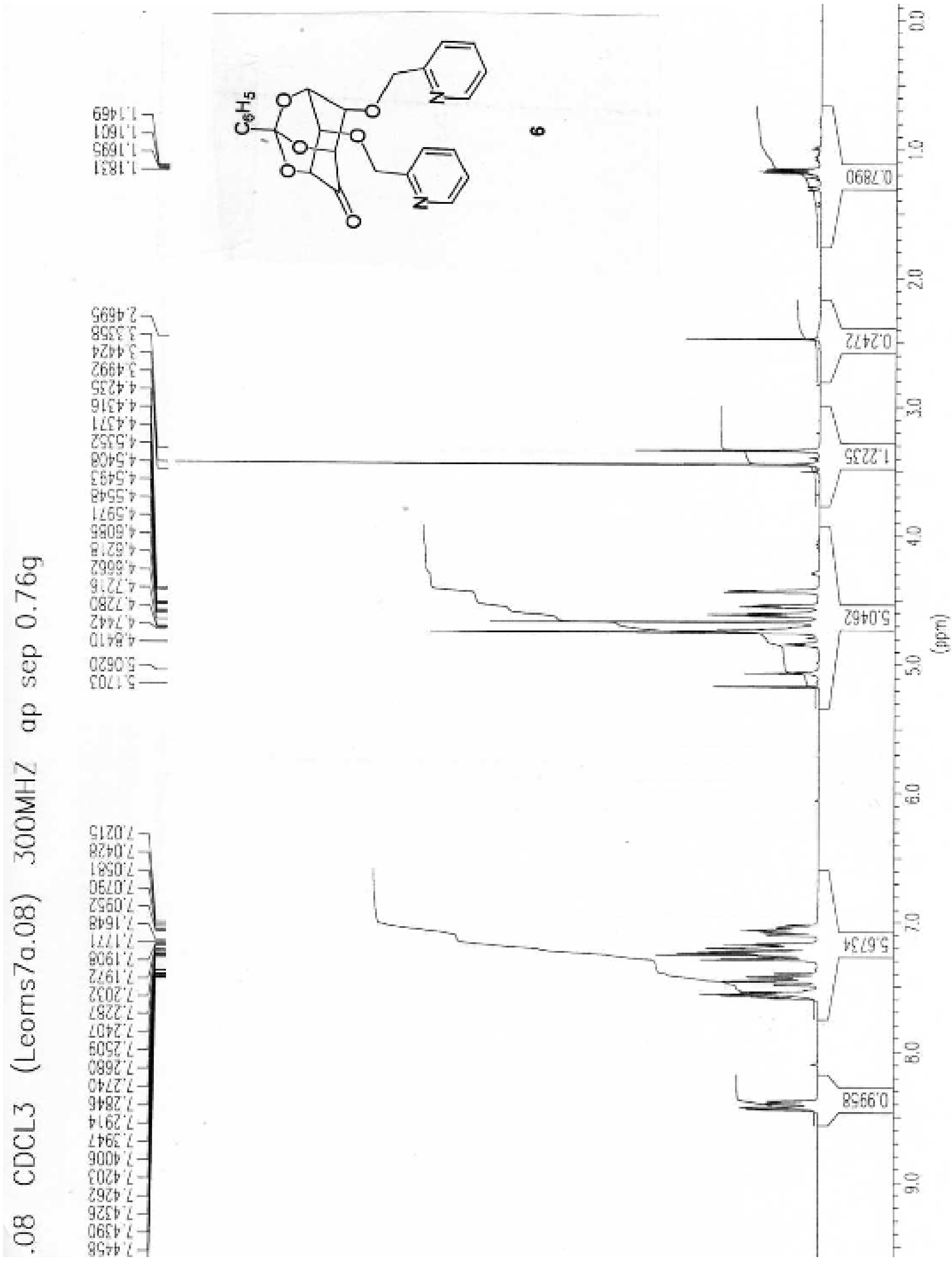

.08 CDCL3 (Leoms7a.08) 300MHZ ap sep 0.76g
C6H5
6
(ppm)
0.7890
0.2472
1.2235
5.0462
5.6734
0.9958

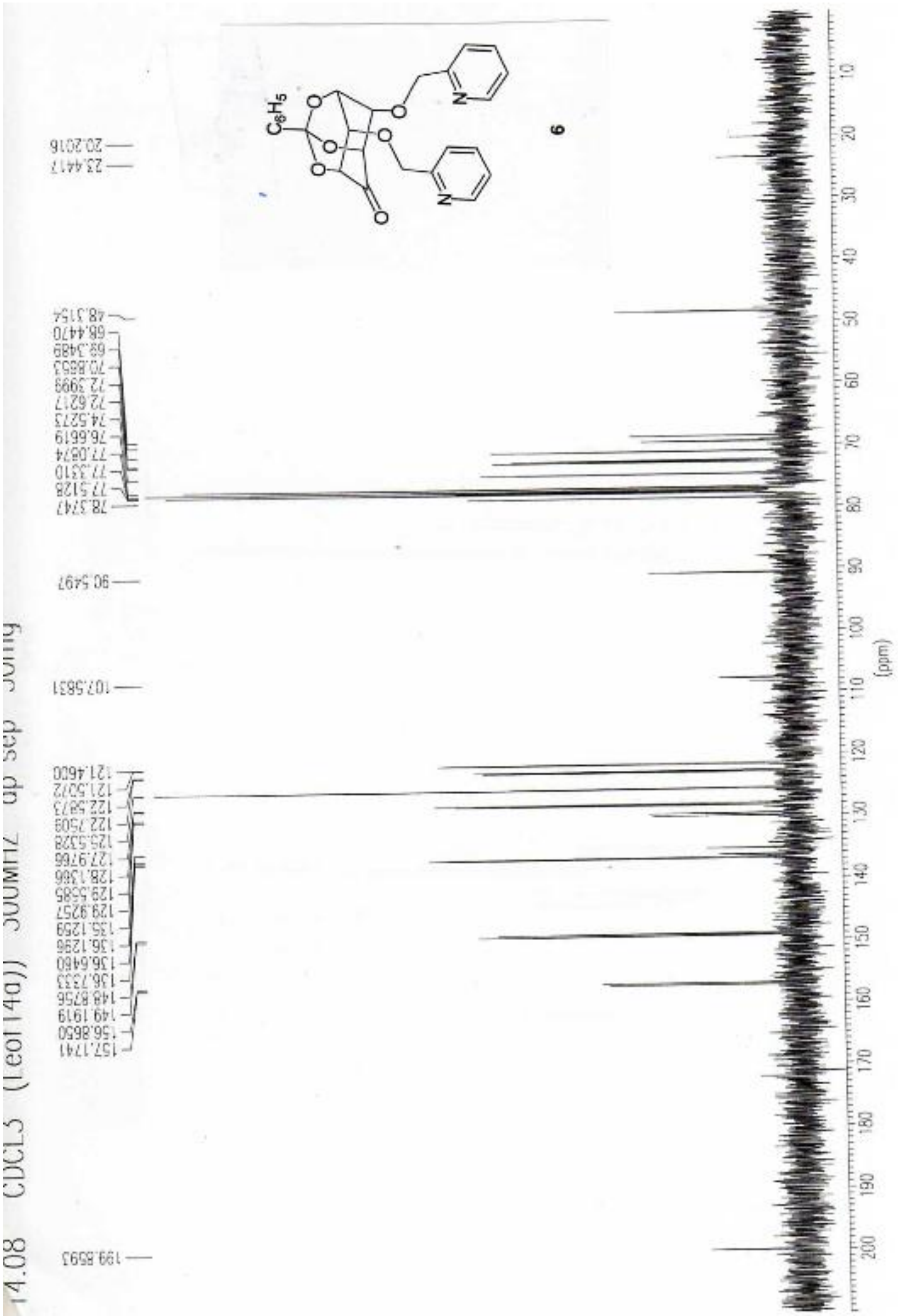
14.08 CDCL3 (Leof14a)) 500MHz dp sep 30mg
20.2016
23.4417
48.3154
66.4470
69.3489
70.8653
72.3999
72.6217
74.5273
76.6619
77.0674
77.3310
77.5128
78.3747
90.5497
107.5831
121.4600
121.5072
122.5873
122.7503
125.5328
127.9766
128.1366
129.5585
129.9257
135.1259
136.1296
136.6460
136.7333
148.8756
149.1919
156.8650
157.1741
199.8593
C_6H_5
6
(ppm)

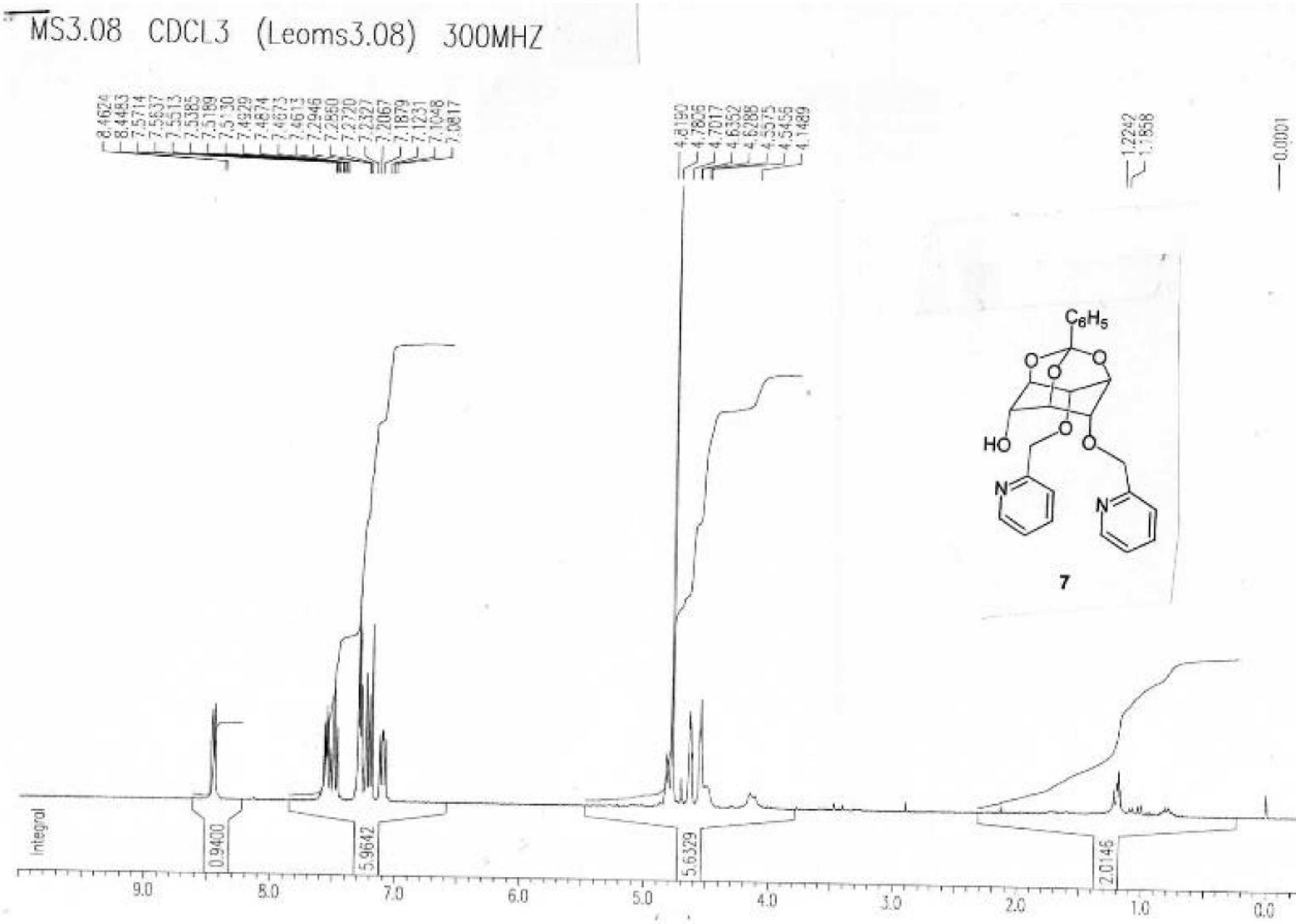

MS3.08 CDCL3 (Leoms3.08) 300MHZ
8.4624
8.4483
7.5714
7.5637
7.5513
7.5385
7.5189
7.5130
7.4929
7.4874
7.4673
7.4613
7.2946
7.2560
7.2720
7.2327
7.2067
7.1879
7.1231
7.1048
7.0817
4.8190
4.7806
4.7017
4.6352
4.6288
4.5575
4.5456
4.1489
1.2242
1.1558
0.0001
C_6H_5
HO
O
N
7
Integral
0.9400
5.9642
5.6329
2.0146
9.0
8.0
7.0
6.0
5.0
4.0
3.0
2.0
1.0
0.0

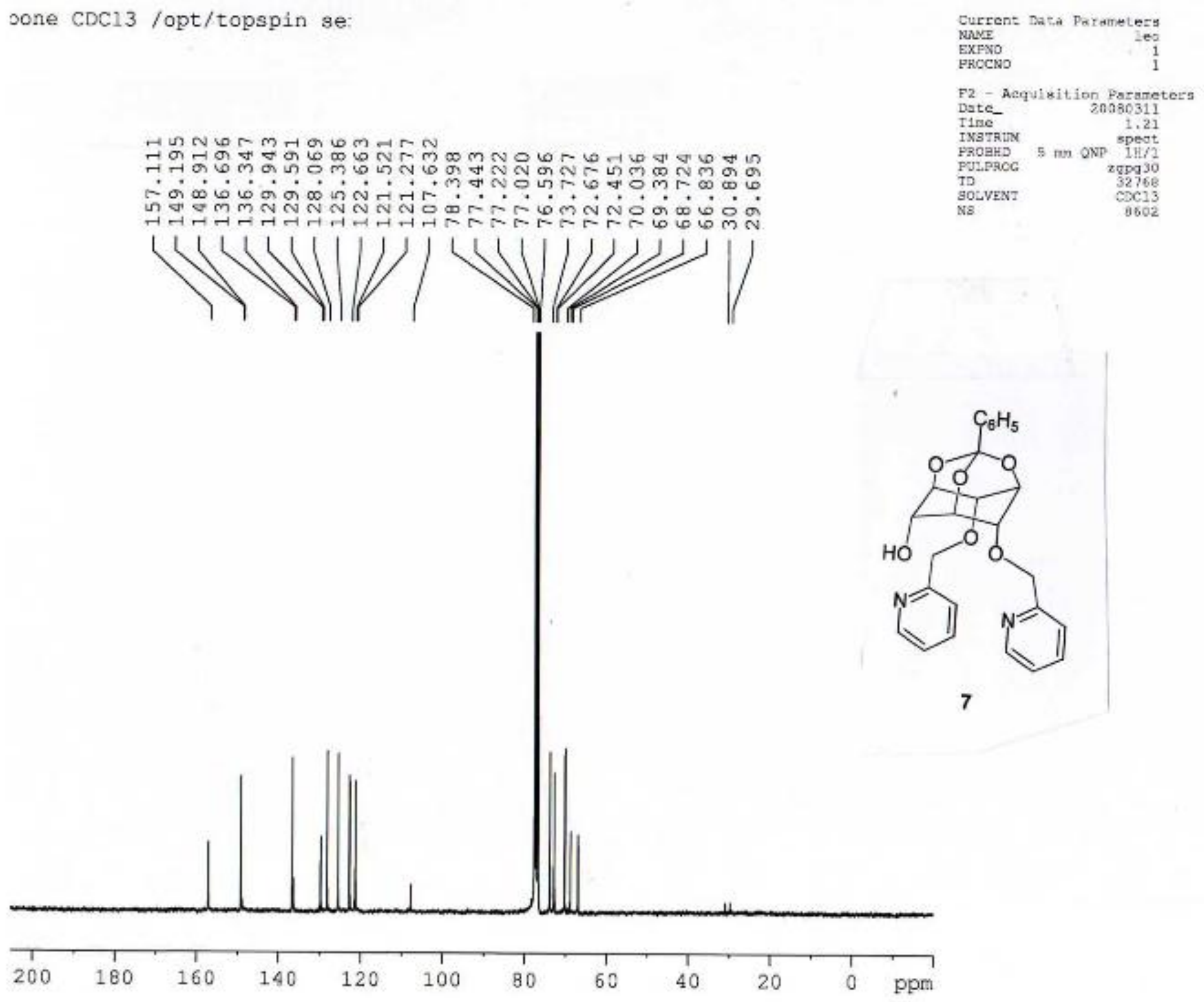

oone CDCl3 /opt/topspin se:
157.111
149.195
148.912
136.696
136.347
129.943
129.591
128.069
125.386
122.663
121.521
121.277
107.632
78.398
77.443
77.222
77.020
76.596
73.727
72.676
72.451
70.036
69.384
68.724
66.836
30.894
29.695
Current Data Parameters
NAME leo
EXPNO 1
PROCNO 1
F2 - Acquisition Parameters
Date_ 20080311
Time 1.21
INSTRUM spect
PROBHD 5 mm QNP 1H/1
PULPROG zgpg30
TD 32768
SOLVENT CDCl3
NS 8602
C6H5
HO
O
N
7
200
180
160
140
120
100
80
60
40
20
0
ppm
